# Nuclear Receptor 4a3 (Nr4a3) Regulates Murine Mast Cell Responses and Granule Content

**DOI:** 10.1371/journal.pone.0089311

**Published:** 2014-02-20

**Authors:** Gianni Garcia-Faroldi, Fabio R. Melo, Dennis Bruemmer, Orla M. Conneely, Gunnar Pejler, Anders Lundequist

**Affiliations:** 1 Swedish University of Agricultural Sciences, Department of Anatomy, Physiology and Biochemistry, BMC, Uppsala, Sweden; 2 Saha Cardiovascular Research Center, University of Kentucky College of Medicine, Wethington, Kentucky, United States of America; 3 Department of Molecular and Cellular Biology, Baylor College of Medicine, Houston, Texas, United States of America; University of Muenster, Germany

## Abstract

Nuclear receptor 4a3 (Nr4a3) is a transcription factor implicated in various settings such as vascular biology and inflammation. We have recently shown that mast cells dramatically upregulate *Nuclear receptor 4a3* upon activation, and here we investigated the functional impact of Nuclear receptor 4a3 on mast cell responses. We show that Nuclear receptor 4a3 is involved in the regulation of cytokine/chemokine secretion in mast cells following activation via the high affinity IgE receptor. Moreover, Nuclear receptor 4a3 negatively affects the transcript and protein levels of mast cell tryptase as well as the mast cell’s responsiveness to allergen. Together, these findings identify Nuclear receptor 4a3 as a novel regulator of mast cell function.

## Introduction

Mast cells are important components of the innate and adaptive immune system, most notably taking part in the defense against parasites, bacteria and viruses [1], [2]. However, there are also several detrimental effects associated with mast cells, e.g. allergy and rheumatoid arthritis [1], [2]. The mast cell expresses the high affinity IgE-receptor, FcεRI. FcεRI ecrosslinking, e.g. by an allergen, results in the immediate release of preformed mediators that are stored in secretory granules [3] and also to the activation of transcriptional events, e.g. mediated by NFAT, NFκB and AP-1, leading to cytokine generation and release. Mast cells are broadly divided into mucosal and connective tissue types based on their repertoire of expressed proteases, for instance chymase, tryptase and carboxypeptidase A3 (CPA3) type, though how the expression of these proteases is regulated is poorly understood [1], [2], [4]. Transcriptional regulation involving GATA-1, FOG and MITF is also an essential part of the development of the mast cell into its different subtypes [5], [6], and mast cell development and phenotype is additionally influenced by the cytokine and growth factor milieu in the respective tissues [7], [8].

The nuclear receptor subfamily 4a (Nr4a) encompasses three members, *Nur77/Nr4a1*, *Nurr1/Nr4a2* and *NOR-1/Nr4a3*, which are early response genes reacting to a variety of stimuli such as cytokines, infectious agents, growth factors and cellular stress [9]–[13]. The activities of the Nr4a3 family members are ligand-independent, meaning that once translated they do not require further stimuli to exert their function. However, their activity is influenced by cellular location, which might be regulated by phosphorylation [14]. Members of the Nr4a family partake in many cellular responses, including those affecting the immune system and inflammation as shown by the contribution of Nr4a1 and Nr4a3 to induction of T-cell and macrophage apoptosis [14]–[16]. Moreover, cytokine and chemokine responses are regulated by Nr4a family members in both macrophages and T-cells, lending additional support of their role in immunomodulation [17], [18].

In a previous study where the global impact of bacterial infection on mast cell responses was studied, it was noted that exposure of mast cells to gram-positive Streptococci resulted in a dramatic upregulation of all three Nr4a members, with the most striking increase noted for Nr4a3 [19]. In a subsequent study we showed that Nr4a members, in particular Nr4a3, were strongly induced also by mast cell activation through FcεRI crosslinking [20]. Prompted by these findings, we here investigated the functional impact of Nr4a3 on mast cells. We show that Nr4a3 is required for optimal cytokine generation following antigen-induced mast cell activation. Moreover, we show that the presence of Nr4a3 regulates FcεRI-mediated degranulation, and finally we show that Nr4a3 selectively suppresses the transcript and the resulting protein of the tryptase mouse mast cell protease 6 (mMCP-6; encoded by *Tpsb2*). Together, these findings suggest a hitherto unrecognized role of Nr4a3 in regulating mast cell homeostasis and activation.

## Materials and Methods

This study was carried out in strict accordance with the recommendations in the Guide for the Care and Use of Laboratory Animals of the National Institutes of Health. The institutional animal care and use committee at the University of Kentucky approved all procedures on mice. The animals were maintained and treated according to the guidelines set by IACUC. Euthanasia was carried out using CO_2_ according to AVMA Guidelines for the Euthanasia of Animals.

### Reagents

Polyclonal antibodies against mMCP-6 (encoded by *Tpsb2*), and CPA3 were raised in rabbits [22]. Antibodies from Cell Signaling were purchased to identify NFAT1 (5861), Fyn (4023), Lyn (2796), IKKβ (2370) and IKKα (2682). Rabbit polyclonal antibody towards β-actin (sc-130656) was from Santa Cruz. ELISA kits were from eBioscience (MCP-1, IL-6, IL-13) and Peprotech (TNFα), respectively.

### Cell Culture

Bone-marrow cells from C57BL/6 mice were obtained from femural and tibial bone of wild type (WT) and Nr4a3^−/−^ [21] mice and cultured in DMEM, supplemented with 10% heat-inactivated fetal bovine serum, 60 µg/ml Penicillin, 50 µg/ml Streptomycinsulfate, 2 mM l-glutamine and 10 ng/ml recombinant IL-3 (Peprotech). The cells were kept at a concentration of 0.5–1×10^6^ cells/ml with weekly changes of medium [22].

### Mast Cell Activation

For IgE-induced activation, cells were sensitized with anti-TNP IgE at 1 µg/ml overnight, washed, resuspended in new media and stimulated with TNP-OVA (0.4 µg/ml or indicated concentrations) [22]. Cells were collected at various time points by centrifugation; media and cell fractions were frozen and stored at −20°C. The effect on TNFα, MCP-1, IL-6 and IL-13 release was determined in cell supernatants using ELISA as detailed by the manufacturer.

### β-Hexosaminidase Activity

β-Hexosaminidase activity was measured in solubilized cell pellets and supernatants from resting or activated cells as described [22].

### Cell Lysate

Lysate buffer (200 ml PBS, 2 M NaCl, 0.5% Triton X-100) was added to cell pellets (1×10^6^ cells/pellet) followed by vortexing. The protein concentration in the lysates was determined with the Bradford method using BSA as a standard. The determined protein concentration was used for normalizing the enzyme activity and Western blot protein loading. Samples used for Western Blot analysis were treated with protease and phosphatase inhibitors.

### Enzymatic Activity

Duplicate samples of cell lysate (20 ml) were mixed with 80 ml H_2_O to which 20 ml of a 2 mM solution (in H_2_O) of the chromogenic substrate S-2288 (H-D-Isoleucyl-L-prolyl-L-arginine-p-nitroaniline dihydrochloride; Chromogenix) was added. Changes in absorbance were recorded at 405 nm and used to calculate initial reaction velocities.

### Induction of Cell Death

Triplicates of 1 ml BMMCs (0.5×10^6^ cells/ml) were transferred into individual wells of a 24-well flat-bottomed plate, and were either left untreated or treated with cytotoxic agents: L-Leucyl-L-Leucine methyl ester (LLME, 200 mM), Cycloheximide (CHX, 5 µg/ml) or H_2_O_2_ (0.75 mM) in complete culture medium, followed by incubation for different time periods (as specified in the figure legends). Triplicates of 100 µl of cell suspension were transferred into a 96-well plate and cell viability was measured using CellTiter-Blue reagent (Promega-Invitogen, Carlsbad, CA). The plate was read using a microplate reader (Infinite M200– TECAN) at 560 nm (excitation) and 590 nm (emission).

### Western Blot

Cell lysates were mixed with 4× reducing SDS-PAGE sample buffer and subjected to SDS–PAGE on 12% gels. Thereafter, proteins were blotted onto PVDF membranes, followed by blocking in Odyssey Blocking Buffer diluted 1∶1 in TBS for 1 h. Next, the membranes were incubated with primary antibodies (diluted 1∶500 for mMCP6, diluted 1∶1000 for all others) in Odyssey Blocking Buffer:TBS (1∶1), overnight at 4°C. After washing with TBS/0.1% Tween-20, the membranes were incubated with appropriate secondary antibodies conjugated to IRDye 800 or IRDye 680 (diluted 1∶1000 in Odyssey Blocking Buffer:TBS) for 1 h at room temperature. The membranes were subsequently washed twice in TBS/0.1% Tween-20 and once in TBS before detecting the signal using an Odyssey Infrared Imaging System (Li-Cor Biosciences, 9201-01).

### Quantitative Real Time RT-PCR

Total RNA preparation and quantitative real-time PCR (qPCR) were performed as previously described [23]. For primers used, see Table 1.

**Table 1:** Primers used for qPCR.

Target	Sequence
mouse	
*Hprt, fw* a	5′-GAT TAG CGA TGA TGA ACC AGG TTA-3′
*Hprt, rev* b	5′-GAC ATC TCG AGC AAG TCT TTC AGT C-3′
*Srgn, fw*	5′-GCA AGG TTA TCC TGC TCG GAG-3′
*Srgn, rev*	5′-GGT CAA ACT GTG GTC CCT TCT C-3′
*Tpsb2, fw*	5′-CAT TGA TAA TGA CGA GCC TCT CC-3′
*Tpsb2, rev*	5′-CAT CTC CCG TGT AGA GGC CAG-3′
*Mcpt4, fw*	5′-GAA GCT TCA AAA GAA-3′
*Mcpt4, rev*	5′-GGT TCT GTC ACT CCA-3′
*Cpa3, fw*	5′-TGA CAG GGA GAA GGT ATT CCG-3′
*Cpa3, rev*	5′-CCA AGG TTG ACT GGA TGG TCT-3′

All primers were designed using the software *Primer Express* Version 1.0.

a fw, forward primer,

b rev, reverse primer.

### Statistical Analysis

Statistical analyses using two tailed, Student’s t-test or ANOVA were performed using GraphPad Prism 4. Differences were considered significant if the p-values were 0.05 or less. All experiments were repeated at least three times; representative experiments are displayed.

## Results and Discussion

### Nr4a3 is Required for Optimal FcεRI-induced Cytokine/Chemokine Generation in Mast Cells

**Figure 1:**
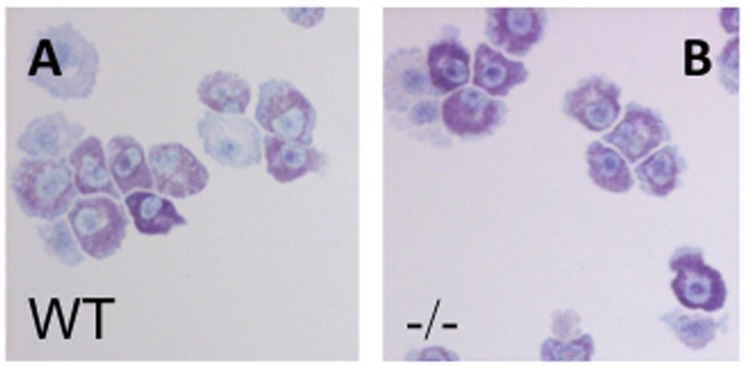
Nr4a3 does not affect mast cell development. (A, B) Toluidine blue staining of WT (A) and Nr4a3^−/−^ (B) mast cells after 4 weeks of culture. Note that the absence of Nr4a3 does not affect the morphology or granular staining of the cells.

To study the influence of Nr4a3 on mast cell function we cultured bone-marrow cells isolated from WT and Nr4a3-deficient mice in the presence of IL-3, which results in maturation of precursor cells into mast cells, i.e. bone marrow-derived mast cells (BMMCs). Toluidine blue staining of four-week old cultures showed no apparent morphological differences between WT and Nr4a3-deficient mast cells (Fig. 1A–B), suggesting that Nr4a3 does not affect mast cell development.

**Figure 2:**
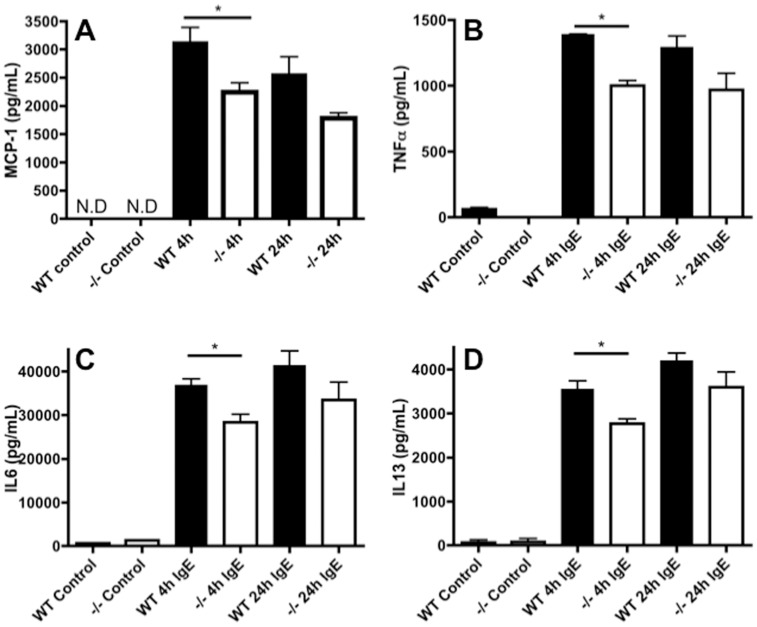
Nr4a3 affects cytokine/chemokine secretion in response to FcεeRI cross-linking. (A–D) WT and Nr4a3^−/−^ mast cells were incubated with TNP-specific IgE over-night followed by activation for 4 hours or 24 hours with TNP-OVA as indicated. Supernatants were analyzed for levels of MCP-1 (A), TNFα (B), IL-6 (C) and IL-13 (D) by ELISA (T-test, n = 4; *p≤0.05). The lack of Nr4a3 is associated with a reduction in cytokine release following FcεRI cross-linking.

In a previous study we found that the *Nr4a3* transcript was potently upregulated following FcεRI crosslinking, suggesting that Nr4a3 might participate in the regulation of this pathway [20]. The events triggered by FcεRI crosslinking include cytokine/chemokine induction as well as degranulation whereby the contents of the mast cell secretory granules (e.g. β-hexosaminidase, proteases and biogenic amines) are released. To explore the potential role of Nr4a3 in regulating these processes we first studied the effect of Nr4a3-deficiency on the secretion of IL-6, IL-13, MCP-1 and TNFα, based on the known importance of these cytokines/chemokines in mast cell responses [1]. As seen in Fig. 2A–D, the absence of Nr4a3 led to a significant reduction in the secretion of the investigated cytokines and chemokines in response to FcεRI crosslinking, thus indicating that Nr4a3 promotes the induction of these factors. In turn, this suggests that Nr4a3 may have a pro-inflammatory role in terms of regulating cytokine/chemokine responses in a mast cell setting. The findings are in line with previous studies in which Nr4a family members have been implicated in the regulation of inflammatory gene expression in macrophages activated through pattern recognition receptors [18].

The canonical NFκB-pathway has been implicated in antigen-induced generation of various cytokines in mast cells [24]. It has been shown that Nr4a-members influence NFκB-signaling by modulating the expression of components of the Inhibitor of nuclear factor-κB-kinase (IKK) complex in macrophages [18]. In mast cells, IKKα and IKKβ have been shown to be important for cytokine generation following antigen activation [24], [25]. Nr4a3-mediated effects on the expression of IKKα and IKKβ could therefore potentially explain the reduced cytokine response in mast cells devoid of Nr4a3. We thus measured the levels of IKKα and IKKβ in mast cells but found that neither the IKKα nor the IKKβ levels were significantly altered due to Nr4a3 deficiency (data not shown). The major pathway for FcεRI-induced IL-13, MCP-1 and TNFα generation in mast cells involves transcription factors belonging to the Nuclear Factor of Activated T cells (NFAT) family [20], [26]. We therefore explored the possibility that the lack of Nr4a3 influenced the protein levels of NFAT1 in mast cells, but could not detect any significant change (data not shown).

**Figure 3:**
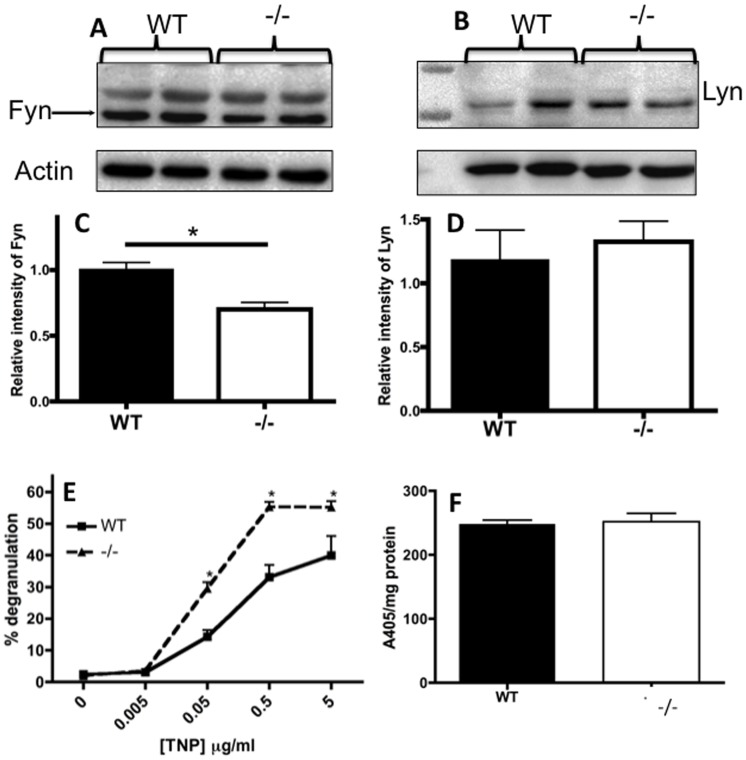
Nr4a3 modulates signaling pathways involved in mast cell degranulation (A–F). (A–B) Whole cell lysates from resting WT and Nr4a3^−/−^ mast cells were prepared and analyzed by Western blot for the Src-kinases Fyn (A) and Lyn (B). The signal was compared against β-actin followed by normalization using the average signal from WT samples set to 1. (C–D) Graphical representation of the densitometric analysis for Fyn (C) and Lyn (D) (T-test, n = 4; *p≤0.05). (E) WT and Nr4a3^−/−^ mast cells were incubated with TNP-specific IgE over-night followed by activation for 30 minutes with increasing amounts of TNP-OVA. Supernatants were collected and were analyzed for β-hexosaminidase activity as a measure of the extent of degranulation. (ANOVA, n = 4; *p≤0.05). (F) Whole cell lysates were prepared and analyzed for their β-hexosaminidase content.

The earliest event following FcεRI crosslinking is activation of the Src-family kinases Lyn and Fyn, which phosphorylate the immunoreceptor tyrosine-based activation motifs of the β and γ subunits of FcεRI [27], [28]. Both Lyn and Fyn are positive regulators of the downstream signaling cascade leading to degranulation and cytokine generation. When determining the total levels of Fyn and Lyn we found that Nr4a3 deficiency was associated with reduced Fyn expression whereas Lyn was unaffected (Fig. 3A–D). In mast cells, Lyn and Fyn both propagate the signal via Syk but Fyn also utilizes pathways involving phosphatidylinositol 3-kinase (PI3K) or Stat5 [29], [30]. Future investigations will determine which signaling pathway is affected by the Nr4a3 dependent reduction in Fyn levels. Nevertheless, the reduction in Fyn levels could be one explanation for the reduced cytokine generation in Nr4a3 deficient mast cells, but a direct effect of Nr4a3 on cytokine transcription cannot be ruled out.

To further assess whether Nr4a3 influences the degree of FcεRI-induced activation, we determined whether the actual extent of degranulation in response to FcεRI crosslinking was affected by the absence of Nr4a3. To this end we measured the release of β-hexosaminidase. Interestingly, in dose-response experiments with increasing concentrations of antigen, Nr4a3^−/−^ mast cells exhibited an increased reactivity to antigen as illustrated by the augmented release of β-hexosaminidase from activated Nr4a3-deficient mast cells compared to their WT counterparts (Fig. 3E). A possible explanation for this finding would be that Nr4a3 affects the production and storage of β-hexosaminidase. However, total β-hexosaminidase activity determinations showed that WT and *Nr4a3* ^−/−^ mast cells were indistinguishable (Fig. 3F). Taken together, these findings thus indicate that Nr4a3 influences processes leading to degranulation.

**Figure 4:**
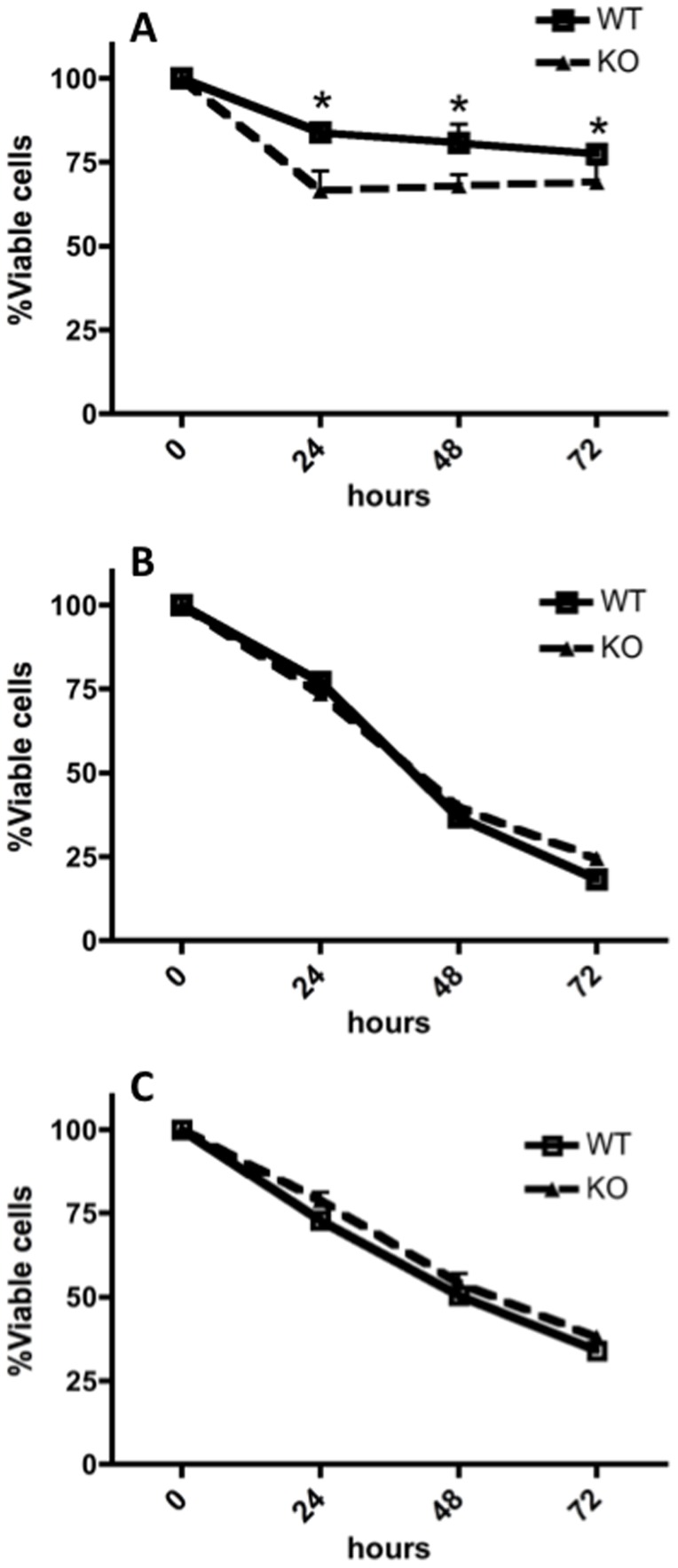
(A–C) Nr4a3-deficient mast cells are more susceptible than WT cells to cell death induced by granule disruption. WT and Nr4a3^−/−^ mast cells were treated with 200 mM LLME (A), 5 µg/ml Cycloheximide (B) or 0.75 mM H_2_O_2_ (C). The viability was determined at the indicated time-points using CellTiter-Blue reagent (ANOVA, N = 6, *p≤0.05).

Previous studies have implicated Nr4a family members, in particular Nr4a1 and Nr4a3, in apoptosis [14]–[16]. It was therefore of interest to investigate whether the absence of Nr4a3 affects apoptosis in a mast cell context. To address this, mast cells were subjected to the pro-apoptotic agents cycloheximide, H_2_O_2_ or L-Leucyl-L-Leucine methyl ester (LLME) followed by measurements of viability [31]–[33]. Interestingly, we found that Nr4a3-deficient mast cells were more susceptible to LLME-induced cell death than were WT cells, whereas no difference in sensitivity to cycloheximide and H_2_O_2_ was seen when comparing WT and *Nr4a3*−/− mast cells (Fig. 4A–C). We have recently shown that tryptase, and other serglycin-dependent proteases, induce cell death when released into the cytosol following exposure to the lysosomotropic compound LLME [32], [34]. Changes in protease levels due to the lack of Nr4a3 could hence explain the difference in susceptibility to LLME-induced cell death.

### Nr4a3 Regulates the Transcription of Serglycin and the Tryptase *Tpsb2*

**Figure 5:**
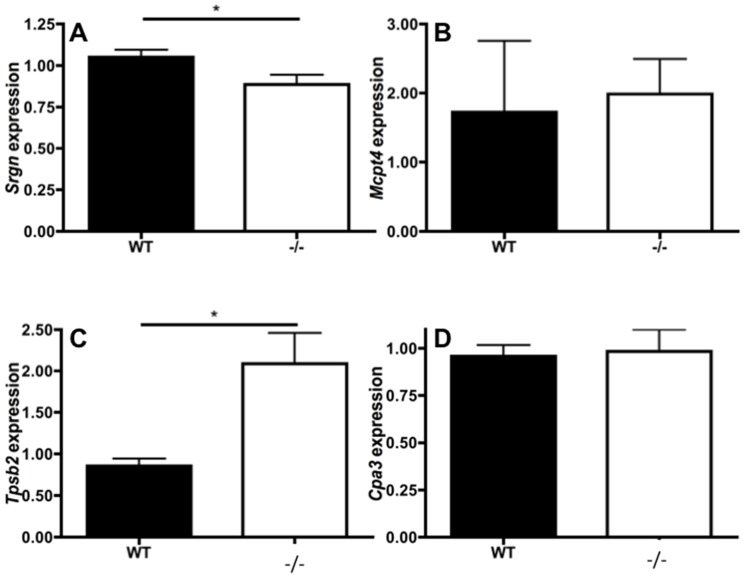
Nr4a3 regulates the transcription of mast cell granule components. Total RNA was extracted from WT and Nr4a3^−/−^ mast cells followed by qPCR analysis for levels of mRNA encoded by *Srgn* (A), *Mcpt4* (B), *Tpsb2* (C) and *Cpa3* (D). *Hprt* was used as house-keeping gene; transcript data were calculated according to DDCT relative to the expression in WT cells (T-test, n = 4; *p≤0.05).

A characteristic feature of mast cells is their secretory granules containing vast amounts of histamine alongside mast cell-specific proteases, the latter requiring serglycin proteoglycan to be stored [35], [36]. To investigate if Nr4a3 influences mast cell function by regulating the mast cell-specific proteases and serglycin proteoglycan, we performed qPCR determinations of transcripts for serglycin (*Srgn*), chymase (*Mcpt4*), tryptase (*Tpsb2*; encoding mMCP-6) and CPA3 (*Cpa3)* in WT and Nr4a3^−/−^ mast cells (Fig. 5A–D) using the primers specified in Table 1. We found that Nr4a3 deficiency led to an ∼2-fold increase in *Tpsb2* transcript levels and to a minor, but significant, reduction of *Srgn* transcript, whereas the expression of *Mcpt4* and *Cpa3* were unaffected.

**Figure 6:**
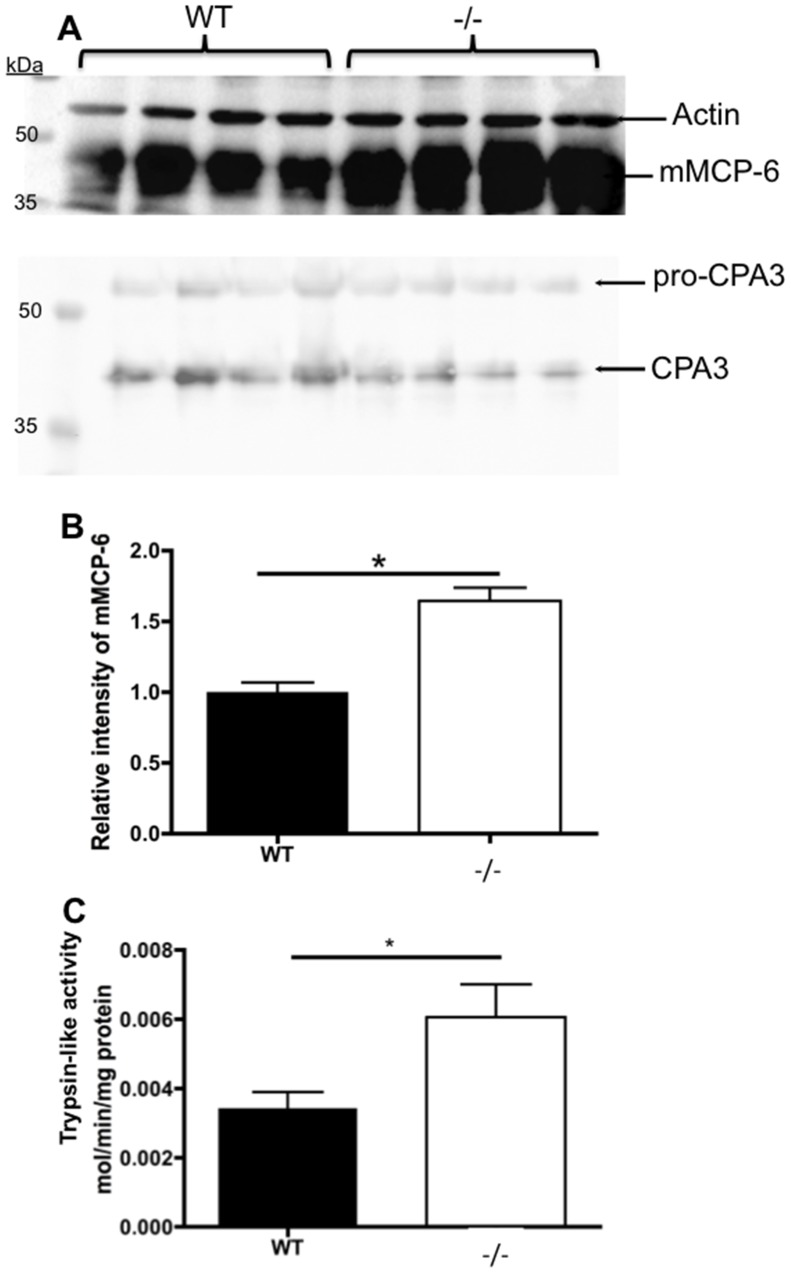
Nr4a3 suppresses the levels of tryptase protein and enzymatic activity. (A) Whole cell lysates from WT and Nr4a3^−/−^ mast cells were prepared and analyzed by Western blot for mMCP-6 and CPA3 as indicated (the upper and lower panels represent individual analyses, performed on the same membrane that was stripped of protein between analyses). The absence of Nr4a3 results in increased levels of mMCP-6 but does not affect CPA3. (B) Quantification of band intensities for mMCP-6 showing a significant increase in mMCP-6 protein in the absence of Nr4a3. (n = 4; *p≤0.05). The level of mMCP-6 and CPA3 proteins were quantified and compared against b-actin followed by normalization using the average signal from WT samples set to 1. (C) Whole cell lysates from WT and Nr4a3^−/−^ mast cells were prepared and analyzed for trypsin-like activity using the chromogenic substrate S-2288. Note that the absence of Nr4a3 results in increased trypsin-like enzymatic activity. (T-test, n = 4; *p≤0.05).

To elucidate if the effects on transcription translate into a change in protein levels we performed Western blot analysis on cell lysates from WT and Nr4a3^−/−^ mast cells (Fig. 6 A–B). In accordance with the mRNA expression analysis, the levels of mMCP-6 protein were significantly higher in *Nr4a3^−/−^* mast cells than in WT counterparts. Moreover, the increase in mMCP-6 mRNA and protein was accompanied by a corresponding increase in the levels of trypsin-like enzymatic activity, as judged by the rate of turn-over of a chromogenic substrate for trypsin-like proteases such as mMCP-6 (Fig. 6 C). Considering that tryptase is one of the most abundant proteins in mast cells [4], [37], the approximately 2-fold increase in transcript and activity represents a quite substantial change. Based on the notion that mast cell proteases promote cell death, it is plausible that the increased susceptibility of Nr4a3-deficient mast cells to LLME-induced cell death is due to their augmented levels of tryptase.

The expression of the mMCP-6 gene (*Tpsb2)* is governed by MITF, PEBP2/CBF and c-jun, which independently or synergistically regulate *Tpsb2* transcription [38]–[40]. Based on our results it is thus plausible that Nr4a3 either acts as a repressor by directly binding to the *Tpsb2* promoter region, or indirectly by negatively influencing the levels of MITF, PEBP2/CBF and/or c-jun. Tryptase is viewed as an important molecule in a number of inflammatory conditions where its presence either is beneficial [41] or detrimental [42], [43]. Thus, the suppressive influence of Nr4a3 on *Tpsb2* transcription may have important consequences for the outcome of inflammatory settings in which tryptase participates.

The present study identifies a novel role for Nr4a3 in regulating mast cell responses, which is summarized in Fig. S1. Intriguingly, our data suggest that although Nr4a3 promotes the secretion of pro-inflammatory cytokines and chemokines, it exerts a negative influence on pathways leading to degranulation responses and the expression of one of the major granule compounds (mMCP-6). Taken together, the differential influence Nr4a3 has on cytokine generation and tryptase levels suggests that Nr4a3 is involved in several regulatory pathways important for mast cell function. It is thus conceivable that Nr4a3 has a role in regulating the balance between different arms of responses induced by mast cell activation, by promoting degranulation-independent events (cytokine/chemokine secretion) in favor of degranulation and expression of certain granule-localized proteases. It is important to stress that mast cell degranulation, although essentially regarded as a pro-inflammatory event, also has important regulatory consequences through the proteolytic inactivation of pro-inflammatory cytokines by proteases released by degranulation [44]–[47]. The present study has focused on how Nr4a3 influences the antigen-induced responses in mast cells and thus its implications for allergies. Based on the findings in the present investigation it is plausible that interfering with Nr4a3 function may augment immediate hypersensitivity reactions as a result of increased mast cell degranulation, whereby mediators such as histamine are released. However, to fully comprehend the influence of Nr4a3 on mast cell function in allergic reactions, additional research in animal models of allergy is warranted.

## Supporting Information

Figure S1 **Summary of the findings presented in this article.** In resting mast cells, Nr4a3 augments transcription of *Fyn* and *Srgn* whereas *Tpsb2* transcription is suppressed. In mast cells activated by FcεRI cross-linking, Nr4a3 promotes the synthesis and release of cytokines but impairs events leading to degranulation.
(TIF)
