# Supplementary figures and images for: Nuclear Receptor 4a3 (Nr4a3) Regulates Murine Mast Cell Responses and Granule Content
Source: PLoS One. 2014 Feb 20;9(2):e89311. doi: 10.1371/journal.pone.0089311 (PMC3930735; doi:10.1371/journal.pone.0089311)

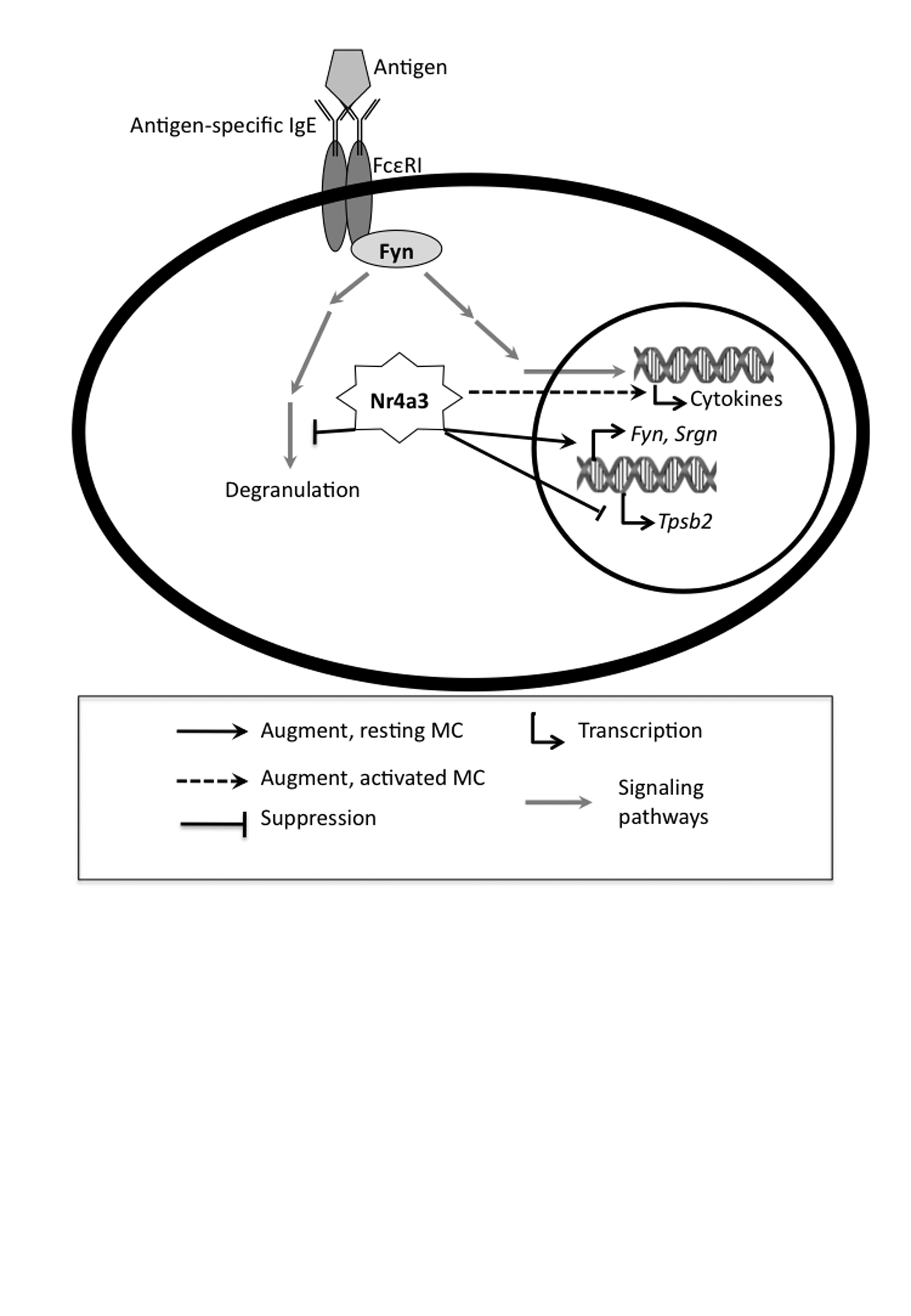

Supplement: Figure S1 — Summary of the findings presented in this article. In resting mast cells, Nr4a3 augments transcription of Fyn and Srgn whereas Tpsb2 transcription is suppressed. In mast cells activated by FcεRI cross-linking, Nr4a3 promotes the synthesis and release of cytokines but impairs events leading to degranulation. (TIF) [file pone.0089311.s001.tif]
